# Navigating the LGB Data Landscape: A Review of Appropriate Secondary Data Sources for Sexuality and Substance Use Research in the UK

**DOI:** 10.3390/ijerph19031329

**Published:** 2022-01-25

**Authors:** Megan Davies, Graham Moon, Clive E. Sabel

**Affiliations:** 1 Department of Public Health, Copenhagen University, 1356 Copenhagen, Denmark; 2 Department of Geography and Environment, University of Southampton, Southampton SO17 1BJ, UK; g.moon@soton.ac.uk; 3 Department of Public Health, Aarhus University, 8000 Aarhus, Denmark; cs@envs.au.dk

**Keywords:** sexuality, substance use, secondary data, survey data, LGB

## Abstract

Research has found that sexual minority individuals are more likely to experience health inequalities and have higher rates of substance use compared with their heterosexual counterparts. This association between sexuality and health outcomes is increasingly being explored using quantitative methodologies within the context of public health, psychology and health geography. Much of this research, however, has relied on primary data, despite the wide availability of secondary sources, mainly survey data, collecting information on sexuality and different types of health outcomes and health risk behaviours, such as substance use. This study reviewed recent surveys in the UK that are appropriate for exploring topics related to LGB populations and substance use behaviours. We carried out a narrative review of secondary data sources in the UK to assess the accessibility and suitability of secondary sources for sexuality and substance use research. We identified eight cross-sectional and two longitudinal surveys that contained both sexuality and substance use data. We summarised the possible applications of each survey and the scope of questions within sexuality and substance use research that could be addressed by each survey. The identification of appropriate surveys in this review can allow researchers to extend the use of secondary data sources in the UK to examine substance use inequalities between sexuality groups, further advancing this key topic.

## 1. Introduction

Sexual minority individuals are more likely than heterosexual individuals to experience adverse health outcomes, inequalities and an increased risk of substance use [1,2,3]. Whilst, originally, this work was predominantly qualitative in nature [4], the shift towards quantitative analyses and taking a more epidemiological approach has meant that there is a need for good-quality quantitative data that allows for the analysis of health inequalities comparatively in lesbian, gay and bisexual (LGB) populations (see table at the end for a glossary of acronyms). The health inequalities experienced by LGB individuals range from increased physical health risks to poorer mental health. Substance use, including illicit drug use, alcohol and tobacco use, have been well documented in this population, and is suggested to be linked to poor mental wellbeing, increased stigma and social norms within the LGB community [5]. Much of the research on substance use in LGB populations has been carried out in the United States, where differing policies on tobacco, for example, means that the key drivers of such inequalities might not be the same as in the UK.

Many of the studies looking at health inequalities and outcomes in LGB populations have utilised primary data, which, while beneficial in that there is often a more representative sample size in the target group, is time consuming. Previous UK Census data does not include sexuality in the questions, and although the 2021 Census will, this may be difficult to access for some time; therefore, one of the most efficient options is using secondary survey data [6]. Secondary data are a valuable tool for researchers, with their advantages including low cost and time efficiency in data collection and typically larger sample sizes than would be collected in primary research, increasing the statistical power [7]. Collecting data on sexuality and substance use can also be difficult, as some individuals might under-report risky behaviours or not respond to particular questions due to the perceived stigma [8]. Whilst this could also apply to the collection of survey data, most funded surveys in the UK are captured face-to-face, which has been shown to increase response rates [9]. The UK is one of the leading countries for relatively open access to secondary sources, hence the focus of this review on UK data sources.

Whilst other sexual and gender minority groups also face health inequalities and unique adverse health experiences and outcomes, trans, intersex and other populations are almost wholly absent from secondary data; thus, our focus here was on LGB populations. Sexuality is often captured in general surveys as part of an inclusion for equality monitoring purposes rather than as a variable for analysis [10]. In many surveys and secondary data sources, sexuality may most commonly be measured by sexual orientation or identity, which can limit and exclude those individuals who do not identify as LGB but may report same-sex attraction or sexual behaviour, and may be at higher risk of engaging in risk behaviours and increased substance use [10,11]. Sexuality, whilst once may have been considered a constant identity, is now increasingly being acknowledged as a fluid and changing concept [12]. The inclusion of sexuality in more surveys and across multiple waves is therefore needed to examine changes in substance use behaviour and other health outcomes within sexuality subgroups, and whether this changes over time or across cohorts. Public health research has also increasingly acknowledged how sexuality is related to other health inequalities between individuals and within communities, with an increased focus on intersectionality.

Whilst previous research has predominantly focused also on individual determinants of substance use in LGB populations, there is an increasing acknowledgement that factors driving health and substance use occurs at both individual and contextual levels [13]. Several studies have included area-level indicators to further understand the risk of substance use inequalities in LGB individuals compared with heterosexual individuals [14,15,16]. It is therefore important that survey data used for sexuality research should include the possibility of exploring these issues beyond individual determinants only. 

The purpose of this study was to highlight secondary data that can be used for sexuality and substance use research in the UK, which is needed to advance this important topic and build on the vast amount of qualitative research on LGB health inequalities [4]. The Office for National Statistics (ONS) recently published a report about sexuality data available in the UK, the majority of which come from administrative data that might be difficult to access for researchers [17]. Only a few social surveys were included that can be used for sexuality research, despite research in the last decade using surveys that were not included in the ONS report [18,19]. Similarly in 2017 Public Health England (PHE) produced a report with UK estimates of the LGB population size from survey data [20]. Whilst this report is useful for accessing sample sizes in specific surveys and surveys that overall include sexuality data, we further extended both these reports in the present study by focussing on secondary survey data that is accessible and appropriate for researchers interested in examining the specific link between sexuality and substance use. We aimed to conduct a narrative review to examine the scope and coverage of sexuality and substance use in accessible secondary data sources in the UK. We also highlight spatial indicators used in each survey that may allow for analysing LGB substance use from both individual and contextual perspectives.

## 2. Methods

We focused on the UK for two reasons: First, the UK holds a rich amount of secondary survey data that can be accessed by researchers, both in the UK and abroad. Whilst other countries have good sources for sexuality and substance use research, such as the Massachusetts Youth Risk Behavior Survey in the United States or the British Columbia Adolescent Health Survey in Canada [21,22], highlighting sources that can be used for this topic outside the UK is beyond the scope of this study. Second, the majority of research on substance use in LGB populations has been carried out in the United States. More UK based studies are needed to understand the context of substance use behaviours in this population in the UK. The focus of this review was also to highlight sources that include both sexuality and substance use data. There are a large number of surveys that include substance use, but we only include them in this review if they also cover sexuality variables. We define substance use as covering illicit drugs, legal drugs that are used recreationally, tobacco, vaping/e-cigarettes and alcohol.

**Figure 1:**
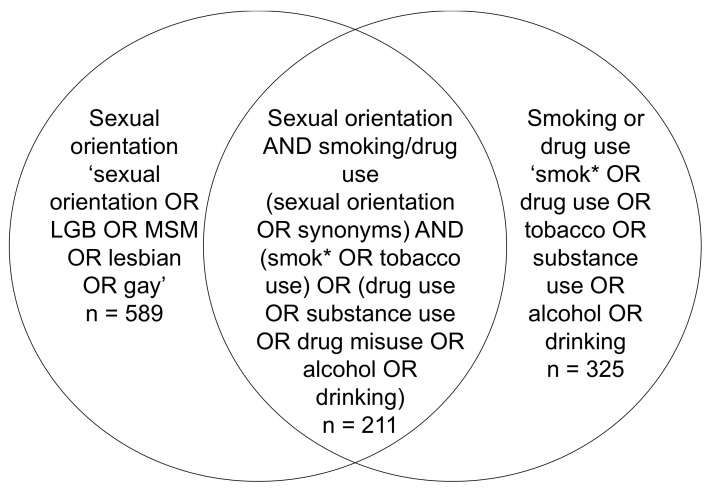
Search terms used to find surveys containing sexuality data, substance use and both.

Based on this criteria, we initially searched bibliographic databases, including Google Scholar and Scopus for UK-based LGB and substance use studies to find out what sources have been used, and a general search for relevant grey literature. Next, we searched for surveys via the UK Data Service (UKDS) discovery search engine, which holds major UK surveys, census data and government-sponsored surveys that are accessible by current researchers. Next, we searched the Medical Research Council (MRC) and Cohort and Longitudinal Studies Enhancement Resources (CLOSER) databases, which hold cohort surveys and longitudinal surveys in the UK, respectively. Separate searches were initially conducted for surveys containing sexuality questions and smoking or drug/alcohol use via the UKDS (see Figure 1). The same search terms were used to search the MRC and CLOSER databases. Sexuality was searched for using synonyms including ‘sexual orientation OR LGBT OR Men who have Sex with Men (MSM) OR sexuality, LGB, gay, lesbian, bisexual’, which returned 589 surveys. Surveys returned from the search included a mix of health surveys, specific sexual lifestyle surveys, household surveys and project-specific surveys. The titles of the surveys were initially assessed for suitability. Many titles represented a different wave of the same survey and were thus considered a duplicate. Surveys containing smoking and drug use questions were also searched for using search terms including ‘smoking OR drug use OR tobacco OR drug misuse OR drug abuse OR drinking OR alcohol’, resulting in 325 surveys. Many surveys overlapped with the sexual orientation search. Again, duplicate surveys and those deemed irrelevant were excluded. Search terms for surveys that included both sexuality and smoking or drug use used a combination of (sexual orientation OR synonyms) AND (smok* OR cigarettes OR tobacco) OR (drug abuse OR drug misuse OR substance use OR alcohol OR drinking), which yielded 211 results. From the other databases, we retrieved 47 surveys from the MRC and 9 from CLOSER.

The inclusion criteria for survey suitability were that surveys included (1) a measure of sexuality and a measure of substance use (illicit drugs, legal drugs for recreational use, alcohol or tobacco); (2) a national sample; (3) openly available in digital form for secondary use or with a clear route for permitted access; (4) individual respondents, i.e., not databases of sexual health clinics or aggregated groups; and (5) surveyed the general population, for example, not prisoners only, and not within an age-specific population, such as youth surveys only or those under 16.

**Figure 2:**
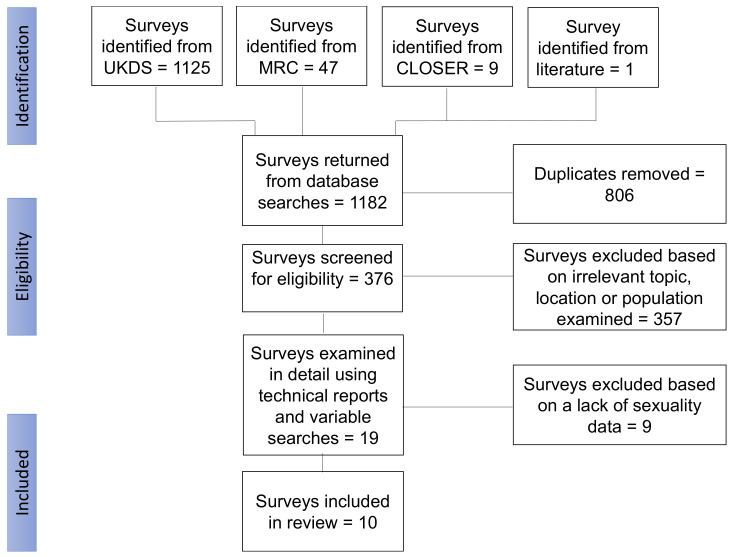
Flow chart of the survey retrieval process.

After removing duplicates and assessing titles and abstracts, 19 surveys were examined in greater detail through reading technical reports and variable searches within documents. Of these, nine were removed due to a lack of appropriate sexuality and/or substance use data, such as asking about attitudes towards substance use rather than personal use (see Figure 2). The 10 surveys retained were based on appropriate data of both sexuality and substance use questions and included a mix of health surveys, specific sexual lifestyle surveys and general household surveys.

## 3. Results

The majority of the results came from the UKDS search, which contained both cross-sectional and longitudinal surveys, and some of the surveys that were returned through the CLOSER and MRC data search engine also appeared in the UKDS search. One survey was identified when reviewing the literature of LGB health inequalities, though this did not appear in any of the searches (Table 1). Of the 10 surveys identified that can be used for sexuality and substance use research in the UK, eight of them were cross-sectional, or repeated cross-sectional surveys, and two were longitudinal (though they were considered in this review as one survey from here onwards, as individuals from the first longitudinal survey series were included in the replacement survey series). All of the surveys addressed sexuality and included questions about substance use, though each survey had different strengths and possibilities for types of analysis and research questions based on other variables that differed between surveys. We also summarised the geographies and spatial indicators that can be used in each survey. We divided the surveys into health surveys, sexuality surveys and general lifestyle surveys, as discussed further below.

### 3.1. Access Conditions

For surveys retrieved through the UKDS, the accessibility is listed on the survey information and abstract. Open data means researchers can access the datasets without registration for the UKDS site. Few surveys on the site are open, and an end-user licence (EUL) requirement is more common. This means data is safeguarded and can only be accessed through registration and accepting the EUL terms. Data that have stricter access conditions, such as obtaining finer level geography variables, can be applied for under Special Licence access. This requires seeking permission from the data owners and justifying the need for a more secure version of the data. Data that is highly detailed or sensitive and confidential can be accessed through the SecureLab, which is an additional application process. Access to surveys that appeared through the MRC and CLOSER databases either need to be contacted directly for access or are signposted to access them through the UKDS. For the European Men who have sex with Men Internet Survey, access is sought directly from the data owners after completing an application form that details a proposed abstract, variables required and whether data are needed for an individual country or multiple countries.

### 3.2. Health Surveys

The Health Survey for England (HSE) and the Scottish Health Survey (SHS) are general health surveys for England and Scotland, whilst the Adult Psychiatric Morbidity Survey (APMS) provides information on mental health in the English population. The UK Biobank is a cohort survey that collects data to examine the link between genetics and health outcomes and diseases, diet and other behavioural health aspects. The General Practitioner Patient Survey (GPPS) covers patient experiences of their GP practice and health services, as well as their health conditions.

The HSE and the SHS both include sexual orientation as part of the questionnaire, although, from 2014, this variable is not available from SHS for researchers from the EUL version from the UKDS. The benefit of these surveys is the broad and detailed health and substance use questions that can be explored in the context of sexuality. Both surveys ask detailed questions about smoking and tobacco use, including different types of smoking behaviours, amount smoked, frequency and cessation, such as motivation to quit. Compared with other surveys, this level of detail could be used to explore sexuality and smoking behaviours in greater detail or with a specific aspect of smoking rather than looking solely at smoking prevalence amongst LGB populations [18]. Alcohol use was addressed in similar detail, including frequency, type of alcohol consumed and questions to assess the level of drinking. Illicit substance use was not addressed in these. The HSE has recently been used to look at various health outcomes, both physical and mental, in LGB populations compared with heterosexuals [23]. Other research has also combined waves of the HSE and SHS, along with other surveys including Understanding Society (US), to look at mental health outcomes and smoking status across sexuality groups [5]. The merging of multiple surveys created a larger sample size, meaning that analysis within subgroups was possible. Depending on the questions and the year examined, these surveys could also be combined to look at Scotland and England together, increasing the sample size of LGB individuals in the survey. Some of the limitations of the HSE and SHS, particularly if used as individual surveys, is the small sample size of LGB individuals, as in 2013, combining the categories of lesbian/gay and bisexual produced a sample size of 2% for HSE and 1.4% for SHS. This could prevent the examination of individual groups within the LGB population due to small numbers.

The APMS focuses on mental health in the adult population, and this survey would be useful for exploring associations between sexuality and mental health outcomes. Sexual identity in this survey encompasses sexual attraction and feelings, and thus the question goes into more detail than other sexual orientation variables in surveys and may include more individuals. The survey also asks about sexual behaviour, which could allow for comparisons and is more inclusive when looking at health outcomes for sexual minority individuals. Substance use was addressed in broad detail, looking at tobacco consumption, alcohol use behaviour and illicit substance use. The APMS includes questions looking at different mental health outcomes and also adverse life experiences. Previous research has used the APMS to explore mental health amongst non-heterosexual individuals overall [24]. Between 5 and 6% of men and women reported being non-heterosexual in the 2007 survey. The exact percentage depended on the wording of the question, as one study found variation in the reporting of same-sex identity if the question asked whether participants were homosexual or gay/lesbian [25]. Therefore, the APMS could allow for a more detailed analysis comparing sexuality groups and different types of substance use behaviour between groups.

The GPPS focuses on patient user experiences at GPs in England, but also asks about socio-demographic information and health questions, such as mental and physical health conditions. The benefit of this survey is the large sample size for each wave (approximately 1 million individuals), with variables weighted to represent the national population as much as possible. Sexuality is measured from self-reported sexual orientation, with a sample size of approximately 2.92% of individuals identifying as LGB [20]. Previous studies have used the GPPS to examine how frequently LGB individuals access GPs compared with heterosexual individuals, and to look at inequalities in long-term health conditions between sexual minority groups compared with their heterosexual counterparts [26,27]. The inclusion of smoking by asking individuals if they are never smokers, former, occasional or regular smokers can allow for analysis on smoking status across sexuality groups. Compared with the HSE and SHS, where the sexuality sample size was smaller, more detailed comparisons between groups could be done without needing to merge different waves across surveys, and also more recent waves can be explored compared with the availability of the SHS.

The UK Biobank could be used to explore longitudinal health outcomes in LGB individuals, though this survey collected data in middle-aged individuals, which limits the possibility to generalise to the wider population. The benefit of using the UK Biobank survey is the large sample size of approximately 500,000 individuals. The Biobank has previously been used to examine whether there is an association between same-sex sexual behaviour and genetic variants [28]. The sample size for those reporting same-sex behaviour is 7.2%, which, due to the question being about behaviour rather than identity, gave a higher percentage of individuals than in other surveys. Substance use included in the UK Biobank includes alcohol intake questions and whether individuals feel addicted or physically dependent, as well as questions about the type of alcohol individuals consume. In terms of illicit drug use, a question about addiction to any illicit substance was asked, as well as whether individuals have ever used cannabis and the frequency of cannabis use. The smoking questions are very detailed, asking about a range of smoking behaviour, frequency, age of initiation and cessation for ex-smokers.

#### Spatial Units for Geographical Analysis

Both the SHS and HSE provide an EUL version of the surveys, which contain government office regions (GORs) as the spatial indicator for HSE and health boards for the SHS. The 2011 Census Output Area Classification is available from the 2014 wave onwards. Whilst spatial indicators below the regional level, such as at the local authority (LA) level, are not available, as the sample size for HSE and SHS are not large enough, the surveys both give Index of Multiple Deprivation (IMD) quintiles for lower layer super output areas (LSOAs). Survey clusters are available from selecting areas and assigning anonymised numbers for each cluster. The inclusion of these allows for some level of spatial analysis, though the lack of finer geographic units excludes the possibility of looking at patterns across England and Scotland in more detail than GORs and health boards.

Spatial analysis for the APMS is similar, in that lower-level geographies are not available due to the sample size of the survey. GORs are available for broader analysis across England, and IMD quintiles are also available. Whilst this limits the possibility of looking at more detailed geographical effects, broader senses of space and area characteristics, such as deprivation level, can be used to examine mental health outcomes between sexuality groups. For the Biobank, data is collected from 35 laboratories across England, Scotland and Wales. Geographical information is limited to mainly location at birth and urban–rural indicators of an individual’s home location. The GPPS contains a binary indicator of urban–rural type of place of residence and IMD quintiles, based on their postcode when completing the survey. These can be used to explore the relationship between urban–rural environments and smoking across sexuality groups or the link between deprivation and smoking and sexuality.

### 3.3. General Social and Lifestyle Surveys

General social and lifestyle surveys returned in the search were Understanding Society (US) and the British Household Panel Survey (BHPS), the Annual Population Survey (APS) and the National Survey for Wales (NSW). These surveys typically ask broad questions pertaining to socio-demographic information; detailed employment and income-related questions; and general health information, including physical health, health behaviours and lifestyle questions.

The NSW covers a range of topics, including socio-demographic, employment, transport and health, with waves since 2013. Sexual orientation is addressed in each wave, but is only available until 2019 due to shorter telephone interviews being conducted during the COVID-19 pandemic. Although in the questionnaire, the categories for sexual orientation are separate (i.e., heterosexual, lesbian/gay, bisexual, other) in the dataset, the categories have been collapsed to a binary variable of ‘heterosexual’ and ‘non-heterosexual’, which includes those who identify as others, as well as those who indicated they prefer not to say. The sample size for these individuals in the 2019 wave of the survey was 5.3%. Substance use questions include those on smoking frequency and e-cigarette use, and more detailed questions on alcohol use. Alcohol use questions include frequency and amount of alcohol consumption and regularity, as well as types of alcohol typically consumed. Illicit substances are not included in the survey.

The BHPS and US are the only longitudinal social surveys included that address sexual orientation. Whilst the BHPS was initially an independent longitudinal survey, the survey stopped in 2009, replaced by the US, which included individuals from the BHPS from wave 2. Individuals from the BHPS can, therefore, be longitudinally followed up if they chose to take part in the US. Despite sexual orientation not being included in the BHPS, the data can be linked through participant ID numbers to their sexual orientation answers in the US. However, not all waves of US include sexual orientation, and as it was not included in the BHPS; therefore, it assumes that sexuality is constant rather than fluid, which is a limitation that needs to be considered when proceeding with linking the data [15]. With the first wave of the BHPS starting in 1991, there are also three decades worth of data, which is a benefit over many of the cross-sectional studies available. In addition to the exploration of health outcomes and behaviours, other questions asking about socio-demographic information, such as employment status and type and education qualifications, could allow for studies looking at social outcomes rather than health specifically. LGB sample sizes for men and women based on the aggregation of individuals from waves 3, 5 and 6 were 2.3% and 1.9%, respectively. Analysis of LGB men and women separately is possible, though looking further within these groups might be difficult depending on the type of analysis.

Substance use questions included in the BHPS are limited to smoking only, asking individuals if they have ever smoked and their current use. US has slightly more detailed questions asking about frequency, consumption amount and cessation efforts. Smoking was included in US from wave 2 only, and in waves 3 and 4, smoking was included only as part of the youth survey for individuals aged 16–21 years. Other substance use questions in US asked about drug use in the last 12 months in all waves except for wave 1, but again was included in all waves only as part of the youth survey. Drug use questions included those about cannabis use, solvent use, other illicit drugs and frequency of drug use. Questions about alcohol use included age of first alcohol drink, frequency of drinking, and how many times in the last 4 weeks an individual has been intoxicated. Previous research has used US to look at health outcomes and mental health amongst LGB individuals and substance use amongst LGB youths [29].

The APS has been collected quarterly since 2004 and is available in yearly datasets. This survey looks at employment issues, housing, socio-demographic information and health. Sexuality is measured through a sexual orientation question, which is used for the estimates for LGB individuals in the UK by the ONS from 2016 onwards. Sexual orientation has been included in the APS from 2011, and a weighted variable is available in the dataset, with a sample size of 2.7%. Questions regarding sexual orientation were asked only to those over 16 and those who took the survey in person, and therefore has a smaller number of individuals in this variable compared with others, though a comparison between sexuality groups could still be possible as opposed to looking at LGB as a whole group. In terms of substance use, the APS looks only at smoking behaviour, which limits the possibility of looking at other substance use behaviours and comparisons between types of substances. As this survey is ongoing with recent and frequent waves, analysis of smoking behaviour amongst LGB individuals and amongst sub-groups can still be beneficial.

#### Spatial Units for Geographical Analysis

LA geographies are available with special access for the NSW; however, there may be some caution as to whether there are enough LGB individuals in the sample to analyse at such a lower geography level. Other area-level indicators include the IMD, detailed urban–rural classifications, as well as a binary urban–rural indicator. GORs are used as the spatial indicator for the standard EUL of both the US and the BHPS. However, a Special License version is also available that contains finer detailed country coding and medium-level and low-level spatial indicators, such as LA-level data. Under Secure Access conditions, there is a version containing British National Grid postcode grid references at a 1 m resolution, derived from the ONS National Statistics Postcode Directory. However, this might not be possible to analyse at such a fine level when looking at LGB individuals due to smaller sample sizes. Northern Ireland was only included in 2001, with earlier surveys including only GB. Whilst the APS has detailed geography indicators, such as LAs and rural–urban classifications, due to the smaller sample size of individuals in the sexual orientation variable, the user guide suggests for researchers to analyse sexuality only at the regional level. Collapsing urban–rural classifications to a binary urban–rural variable could also be used to explore broad urban–rural differences.

### 3.4. Sexuality Surveys

In our review, we identified two sexuality survey series that are appropriate for analysing health outcomes and behaviours in sexual minority populations. The National Survey for Sexual Attitudes and Lifestyle (NATSAL) was retrieved through the UKDS, and has three cross-sectional waves. The European Men who have sex with men Internet Survey (EMIS) had two waves collected in 2012 and 2017. Both these surveys focused on questions about sexuality and how individuals identify, sexual attraction and sexual behaviours. Whilst NATSAL looks at sexual lifestyle for all sexualities, including heterosexual individuals, which make it possible to draw comparisons between men and women and across sexualities, EMIS is only for MSM.

As NATSAL looks at all sexual orientations, comparing LGB to heterosexual individuals is possible. Another possibility with using NATSAL is the opportunity to compare cohorts over time and examine changes in sexual lifestyle or behaviour over roughly three decades. The survey includes questions about both heterosexual and same-sex sexual activity and sexual attraction, extending beyond other surveys that ask only about sexual identity. NATSAL also asks questions including incidences of unsafe sex in the past year and information about sexually transmitted diseases and HIV testing, attitudes to sex and self-perceived risk of HIV. Other health risk behaviours, such as alcohol, smoking and substance use, were also included. Current smoking behaviour and frequency were included, as well as where individuals were ex-smokers. Alcohol use was addressed in greater detail asking about frequency and how often individuals drink over the recommended weekly amount of alcohol. Detailed illicit substance use was addressed through questions about when specific drugs were last taken or whether they were used in the last four weeks, and whether individuals have ever tried cannabis or injected drugs. Previous research has used NATSAL to look at different health outcomes and substance use between sexual minority groups [30,31]. The sample size of LGB individuals in wave 3 of NATSAL was higher than the UK estimate from the APS, at 4.9%. For sexual behaviour, the percentage of individuals reporting same-sex behaviour gave a higher estimate than sexual identity, at 11.6%, whilst individuals who reported any same-sex attraction was even higher at 18%. Whilst individual categories are still relatively small compared with the number of heterosexual individuals, or those who report opposite-sex sexual behaviour or opposite-sex attraction only, careful statistical analysis can allow for exploration across individual sexuality groups, which is important in adding to the current narrative of LGB research within the UK.

Whilst EMIS is beneficial for examining behaviours in MSM populations, there are no opportunities for comparisons with heterosexual populations or drawing comparisons between sexual minority women. EMIS focuses mainly on sexual risk behaviours, including questions about unprotected sex and visits to venues often used for sexual activity, but also includes questions about other health risk behaviours, including alcohol and substance use. Questions about types of illicit drug use and whether individuals have ever used specific types of drugs are included, as well as about injecting drug use and whether they have ever shared needles. Research has explored both individual countries within the dataset and comparisons across European countries in terms of substance use [32,33]. Current alcohol use and frequency of alcohol consumption were included. For smoking, there is only one question about tobacco consumption in general rather than specific smoking behaviours. There are also demographic questions that can allow for more general exploration. EMIS asked similar questions to NATSAL, including sexual risk behaviours, such as unprotected anal sex, visits to gay clubs, sex parties and gay saunas. Knowledge of HIV and HIV testing was included, as well as knowledge about condom safety.

Similar to NATSAL, EMIS allows for the investigation of drug use and tobacco consumption associated with sexuality or sexual risk behaviours. Whilst EMIS includes only MSM, the survey may be able to be paired with NATSAL by looking at questions that are the same; thus, MSM data can then be compared with heterosexual and other sexual minority groups. Some research has compared estimates and probabilities of different questions and outcomes from NATSAL and EMIS and compared the wording of questions, which would be useful for future researchers who might want to link the data [34].

#### Spatial Units for Geographical Analysis

NATSAL measured geographical differences across Great Britain (GB) using GORs to identify the region a participant lived in. NATSAL included urban-rural indicators, with one variable that looked at England and Wales and another that looked at Scotland only. In addition, NATSAL contained ONS urban–rural indicator variables and Output Area Classification codes. Area-level deprivation was also provided by IMD quintiles. The 2011 Area Classifications cover output areas and LA districts. The EUL version of the survey used GORs as units of analysis, whilst a Secure Access version contained LA districts, Area Classification for Output Areas Subgroups and IMD quintiles.

Spatial indicators in EMIS start at the European country level and then the subregions of Europe. Then, within each country, the location is refined further to county areas. In addition, area typologies are asked for in the survey, asking participants about urban–rural indicators and how they would describe the size of the area they live in. For example, very big cities or towns were described as having a population of a million or more people, whilst a village or an area in the countryside included a population size of less than 10,000 people. Therefore, it is possible to look at the type of geography and the link between different substance use outcomes amongst MSM individuals.

## 4. Discussion

This review aimed to highlight sources of secondary data available for sexuality and substance use research in the UK. We identified appropriate secondary sources through a narrative review process to assess the possibility of using each survey for further research looking at both sexuality and substance use behaviours. We aimed to identify secondary sources through a rigorous retrieval and review process of UK databases and other sources through relevant papers and grey literature, ensuring the quality of data sources, whilst reviewing the accessibility and suitability of each survey based on our knowledge of sexuality and substance use research in the UK. We focused on quantitative surveys in this review and, based on a review of abstracts and other documentation, we found that 10 (9 when BHPS and US were counted as one) surveys were appropriate to use for UK sexuality and substance use research. Each of the surveys provides an opportunity to either compare sexuality groups looking at different substance use behaviours and/or the link with other health outcomes in the UK, or to examine outcomes and behaviours in more detail within sexuality groups, such as MSM. Much of the research on sexuality in the UK has relied on primary data or looked primarily at sexuality survey data. The inclusion of sexuality measures in other health and social surveys allows for a wider exploration of issues in this population and provides more options for data access on this topic.

We identified surveys that are suitable for both longitudinal and cross-sectional analyses, as well as the opportunity to examine cohort effects. The shift of sexuality research in the UK from qualitative methodologies and the previous focus on the HIV/AIDS crisis to more quantitative and epidemiological approaches is in line with research emerging from North America in this context [4]. Therefore, it is important for researchers looking at UK LGB issues to know what is available and how we can maximise the use of available secondary data. Each survey has potential benefits and different possibilities to examine LGB issues and substance use inequalities in the UK. Many studies have established the link between an increased risk of poor mental health and substance use issues and identifying as LGB. The surveys discussed in this review allow for the exploration of this in more detail, such as looking across sexual minority groups; using different measures of sexuality, such as sexual behaviour or attraction; and examining possible contextual effects, as well as individual determinants.

### Strengths and Limitations

This review is the first to identify secondary sources of data that are suitable for research on sexuality and substance use in the UK. A similar, though less detailed, review in the ONS highlighted some possible sources that researchers could use, but some surveys that would be useful were not included in that report, such as EMIS [17]. This report covers survey and administrative data on sexual and gender minority data that are available in the UK. The ONS (2020) report makes an argument for the inclusion of sexual orientation in the UK census, which would allow for more generalisations to the UK population and more detailed geography indicators. The surveys discussed in this review can be used by researchers to generate more secondary data papers in sexuality and substance use research. Additionally, there is scope for particular research questions to be identified from the summary of each survey and highlighting which surveys would be most appropriate for particular questions or the application of specific analytical methods.

Limitations of this review are that, first, whilst we have endeavoured to highlight sources that include both substance use and sexuality data, some surveys that include these may not have appeared through our review process. Recent waves of the surveys we reviewed might also have changed their questions or format due to the COVID-19 pandemic, though, at the time of this report, we have ensured our knowledge is up to date. Second, some of the surveys listed had small LGB sample sizes that could make analysis that involves separating groups out or looking at interaction effects more difficult. Finally, Public Health England (2017) produced a report estimating the LGB population size in the UK from available data and surveys [20]. The report summarises surveys and data in the UK that includes LGB questions or samples and lists the most recent wave available and LGB sample size. Similar to the ONS (2020) report, the surveys included in the PHE report that were excluded from this review were omitted due to the survey either being for a specific population or not including questions on substance use in addition to sexuality. However, our review extends the previous reports via the inclusion of substance use measures to further extend this specific topic within sexuality research rather than highlight surveys that only include sexuality data. Finally, this review is limited to discussing the context of sexuality research in the UK and highlighting UK-specific secondary sources. Although EMIS is European-wide and a multi-country comparison is possible, researchers can request a single-country-specific dataset from this survey; therefore, we reviewed this survey based on using the data from the UK only. The focus of the UK in this review means other sources outside the UK that could be used for sexuality and substance use research were not explored, as this was beyond the remit of this study.

## 5. Conclusions

Secondary LGB data in the UK offers the opportunity for researchers to gain access to large sample sizes that are more time and cost efficient. The types of surveys identified in this review each offer different benefits for sexuality researchers. Health surveys offer the opportunity to look at detailed health outcomes and substance use in fine detail in the LGB population compared with the heterosexual population. Household surveys often have larger samples sizes, and the longitudinal nature of the BHPS and US allows us to trace individuals back to 1991 to look at long term changes in outcomes and substance use behaviours in LGB populations. Finally, sexuality surveys can allow for the examination of differences within sexuality groups and look at different measures for sexuality and the link with substance use. This review allows researchers to maximise the use of current secondary data and surveys to further advance quantitative sexuality and substance use research in the UK. Highlighting the accessibility and possible applications of each survey can extend previous research on sexuality and substance use, which is needed to further understand the complex relationship between greater substance use and identifying as LGB.

## Figures and Tables

**Table 1:** Accessibility, background and variables available for each survey.

Survey	Accessibility and Year	Background and Design	Types of Sexuality Variables Available	Types of Substance Use Covered	Other Considerations
Adult Psychiatric Morbidity Survey (APMS)	Available through the UKDS, years 2007 and 2014 are the latest available	This survey aims to give insight into psychiatric disorders, including mood disorders, substance use disorders, personality disorders and anxiety disorders in adults over 16 in England. The methodology used a multi-stage stratified sample of individuals in private households, with one adult randomly selected from households with more than one adult.	Self-reported sexual orientation and past sexual behaviours	Tobacco consumption, alcohol use and illicit substances	Survey looks at England only
Annual Population Survey (APS)	Quarterly waves since 2004	This survey encompasses variables from the labour force survey, which measures employment and unemployment in the UK population .Data is collected quarterly from random households. A rotational sampling design is used, where households, not people, are selected for five consecutive quarters. Data is available in annual waves, containing quarterly datasets for that year.	Self-reported sexual orientation	Smoking	
British Household Panel Survey/Understanding Society (BHPS/US)	Available through the UKDS. BHPS wave available from 1991 until 2009, understanding Society continued the sample from wave 2; latest available wave is for 2018	The BHPS and US are general household surveys The sample design for BHPS used a stratified cluster design, with 250 PSUs in GB, with all adults interviewed in a household. More households and boost samples were added in later waves. The first wave of BHPS in 1991 was 9092 adults interviewed within households. The former BHPS sample was included from wave 2 of US, with those who were still part of BHPS at wave 18 and gave consent to being contacted for taking part in US. US also had a general population sample, which again used postcode sectors as PSUs, where addresses were then selected from these.	Self-reported sexual orientation	Smoking only in BHPS. Smoking, alcohol, both illicit and legal recreational drug use in US	Sexual orientation available in waves 3 and 5 of Understanding Society only
European Men who have sex with men Internet Survey (EMIS)	Accessible through the data owner at the London School of Hygiene and Tropical Medicine; two waves in 2010 and 2017	Two cross-sectional waves. EMIS was an online-administered survey, translated into 25 languages. EMIS was accessible in 38 countries, though the UK-only data was made accessible from the data owner at the London School of Hygiene and Tropical Medicine. EMIS was promoted through social networking sites, blogs, NGO websites and via posters in gay venues. Promotion through the website was either paid or unpaid, depending on the agreement.	Sexuality variables were captured through self-reported sexual identity, past sexual behaviour and sexual attraction	Tobacco consumption, alcohol use, both illicit and legal recreational drug use	Only data on men who have sex with men; women and heterosexual men were excluded
Health Survey for England (HSE)	Accessible through the UKDS, with frequent waves since 1991; latest available wave is from 2018	This survey was administered to adults over 16 to identify general health issues amongst the English population, including physical health, mental health and health risk behaviours. This is a cross-sectional survey repeated annually. The design is a multi-stage stratified random sample collected from primary sampling units. The survey was collected from a mix of completed face-to-face interviews, self-completed questionnaires and a nurse visit to collect biometric data.	Self-reported sexual orientation	Smoking, tobacco, e-cigarettes and alcohol	
National Survey for Wales (NSW)	Available through the UKDS; waves since 2013, latest available wave is from 2020	This survey is administered to adults over 16 annually to randomly selected households, with one adult randomly selected from each household, corresponding to approximately 12,000 individuals each year.	Self-reported sexual orientation	Smoking, e-cigarettes and alcohol use	Sexual orientation included only until 2019 due to a shorter telephone survey in 2020 due to the COVID-19 pandemic
National Survey of Sexual Attitudes and Lifestyle (NATSAL)	Three waves from 1990–2010	NATSAL is a survey about sexual behaviours in Great Britain that has been widely used in research and to inform sexual health interventions and programmes NATSAL used multi-stage, clustered and stratified probability design, whereby an individual was selected at random from addresses that were selected within primary sampling units.	Sexuality variables captured through self-reported sexual identity, past sexual behaviour and sexual attraction	Smoking, alcohol use, both illicit and legal recreational drug use	
Scottish Health Survey (SHS)	Started in 1995, with a wave in 1998 and 2003, then annual waves since 2008	This survey was administered to adults over 16 to identify general health issues amongst the Scottish population including physical health, mental health and health risk behaviours. This is a cross-sectional survey repeated annually. The design is a multi-stage stratified random sample. The addresses were drawn by the Scottish Government since 2012. The survey was collected from a mix of completed face-to-face interviews and self-completed questionnaires.	Self-reported sexual orientation	Smoking, tobacco, e-cigarettes and alcohol use	Sexual orientation variable not available from 2014 due to confidentiality concerns; looks at Scotland only
The General Practitioner Patient Survey (GPPS)	Access through contacting survey team at https://www.gp-patient.co.uk accessed on 20 January 2022	Cross-sectional survey with annual waves since 2007. A sample of eligible patients (had a valid NHS number, registered with a GP practice for at least 6 months before, registered with a GP practice in England, aged 16 and over) from practices that opted in to receive the survey were sent invites.	Self-reported sexual orientation	Smoking	Looks at GP practices in England only
UK Biobank	Accessible through the UK Biobank website. Two time periods of 2006–2010 and a follow up of 2011–2012 are available	Longitudinal survey looking at the link between diet and multiple health outcomes in middle-aged individuals across the UK. Individuals registered with the NHS aged 40–69 living in a certain proximity to assessment centres were invited to take part.	Sexual behaviour only—number of same-sex sexual partners during lifetime	Smoking, alcohol use and illicit drug use	Costs associated with access, which might not be available to all researchers

## Data Availability

Not applicable.

## Glossary of Terms

APMS	Adult Psychiatric Morbidity Survey
APS	Annual Population Survey
BHPS	British Household Panel Study
CLOSER	Cohort and Longitudinal Studies Enhancement Resources
EMIS	European Men Who Have Sex with Men Internet Survey
EUL	End user licence
GOR	Government office regions
GPPS	General Practitioner Patient Survey
HSE	Health Survey for England
LA	Local authority
LGB	Lesbian gay and bisexual
LSOA	Lower layer super output area
MRC	Medical Research Council
MSM	Men who have sex with Men
NATSAL	National Survey of Sexual Attitudes and Lifestyle
NSW	National Survey for Wales
ONS	Office for National Statistics
PHE	Public Health England
SHS	Scottish Health Survey
UKDS	UK Data Service
US	Understand Society
